# Age-Related Intestinal Dysbiosis and Enrichment of Gut-specific Bacteria in the Lung Are Associated With Increased Susceptibility to *Streptococcus pneumoniae* Infection in Mice

**DOI:** 10.3389/fragi.2022.859991

**Published:** 2022-03-18

**Authors:** Rachel H. McMahan, Holly J. Hulsebus, Kevin M. Najarro, Lauren E. Giesy, Daniel N. Frank, David J. Orlicky, Elizabeth J. Kovacs

**Affiliations:** ^1^ Department of Surgery, Division of GI, Trauma and Endocrine Surgery, and Alcohol Research Program, Burn Research Program, University of Colorado Denver, Aurora, CO, United States; ^2^ GI and Liver Innate Immune Program, University of Colorado Denver, Aurora, CO, United States; ^3^ Immunology Graduate Program, University of Colorado Denver, Aurora, CO, United States; ^4^ Department of Medicine, Division of Infectious Diseases, University of Colorado Denver, Aurora, CO, United States; ^5^ Department of Pathology, University of Colorado Anschutz Medical Campus, Aurora, CO, United States

**Keywords:** aging, neutrophil, microbiome, pneumonia, gut-lung axis, innate immunity

## Abstract

The portion of the global population that is over the age of 65 is growing rapidly and this presents a number of clinical complications, as the aged population is at higher risk for various diseases, including infection. For example, advanced age is a risk factor for heightened morbidity and mortality following infection with *Streptococcus pneumoniae*. This increased vulnerability is due, at least in part, to age-related dysregulation of the immune response, a phenomenon termed immunosenescence. However, our understanding of the mechanisms influencing the immunosenescent state and its effects on the innate immune response to pneumonia remain incomplete. Recently, a role for the gut microbiome in age-specific alterations in immunity has been described. Here, we utilized a murine model of intranasal *Streptococcus pneumoniae* infection to investigate the effects of age on both the innate immune response and the intestinal microbial populations after infection. In aged mice, compared to their younger counterparts, infection with *Streptococcus pneumoniae* led to increased mortality, impaired lung function and inadequate bacterial control. This poor response to infection was associated with increased influx of neutrophils into the lungs of aged mice 24 h after infection. The exacerbated pulmonary immune response was not associated with increased pro-inflammatory cytokines in the lung compared to young mice but instead heightened expression of immune cell recruiting chemokines by lung neutrophils. Bacterial 16S-rRNA gene sequencing of the fecal microbiome of aged and young-infected mice revealed expansion of *Enterobacteriaceae* in the feces of aged, but not young mice, after infection. We also saw elevated levels of gut-derived bacteria in the lung of aged-infected mice, including the potentially pathogenic symbiote *Escherichia coli.* Taken together, these results reveal that, when compared to young mice, *Streptococcus pneumoniae* infection in age leads to increased lung neutrophilia along with potentially pathogenic alterations in commensal bacteria and highlight potential mechanistic targets contributing to the increased morbidity and mortality observed in infections in age.

## Introduction

The global population is aging and by 2030 it is predicted that 1 out of 6 people will be over the age of 60 (Beard et al., 2016). Providing care for this population presents a significant challenge as advanced age predisposes individuals to numerous diseases. In particular, advanced age is a major risk factor for increased morbidity and mortality after infections, including pneumonia. Indeed, the incidence of pneumonia in the aged population is estimated to be four times that of younger patients (Loeb et al., 1999). Additionally, older adults are nearly 5 times more likely to be hospitalized following infection (Kaplan et al., 2002) and mortality rates can exceed 50% depending on comorbidities or underlying health conditions that may be present (Muder, 1998; El-Solh et al., 2001). The most common cause of community acquired pneumonia in the elderly is *Streptococcus pneumoniae (S. pneumoniae)* (Stupka et al., 2009; Aliberti and Kaye, 2013). As of 2020, 100 distinct serotypes of *S. pneumoniae* have been identified based on capsular polysaccharide structure (Ganaie et al., 2020), with differing levels of infectiousness and severity of disease. One serotype, serotype 3, is generally associated with lower risk of invasive disease in the pediatric population (Brueggemann et al., 2004), but is commonly found in patient isolates from older adults and is linked to higher risk of severe disease and mortality in this group (Luján et al., 2010). Due to the risk of adverse health outcomes following infection, a full understanding of the mechanisms behind this impaired response to this serotype is an urgent clinical question.

While the reasons for the increased susceptibility to infection in the elderly are likely multifactorial, age-related disruptions in the immune response, or immunosenescence, are thought to play a key role (Krone et al., 2014). Immunosenescence describes a complex phenotype of the aging immune system with upregulation of certain immune functions in conjunction with a downregulation of others. For example, increased baseline levels of pro-inflammatory cytokines are seen with aging, a phenomenon that has been termed inflamm-aging (Franceschi et al., 2000). Mice experience inflamm-aging similar to humans and aged mice also have higher basal circulating levels of pro-inflammatory mediators compared to young (Kovacs et al., 2002). Alternatively certain innate immune cell functions appear to be impaired in age, although this varies between studies, and other innate immune cell functions appear to be maintained, or even increased in age (reviewed in (Shaw et al., 2013)). In the case of neutrophils, which are critical for immune control following *S. pneumoniae* infection, *in vitro* studies have demonstrated reduced phagocytic function and inaccurate migration of neutrophils isolated from older adults (Simell et al., 2011; Sapey et al., 2014).

More recently, intriguing data have begun to emerge pointing to a role for the microbiome in age related changes in immunity. While the mechanisms of this age-related dysbiosis are not fully understood, it is thought to occur, in part, due to changes in gut physiology (DeJong et al., 2020). Although complex, in humans, this age-related dysbiosis is characterized by a decrease in *Akkermansia* and *Bifidobacterium* along with an enrichment of *Proteobacteria* (Bosco and Noti, 2021). Furthermore, transfer of fecal microbiome from aged mice into young germ-free recipient mice results in increased gut inflammation, a breakdown of the intestinal barrier and increased systemic inflammation (Fransen et al., 2017). This effect was associated with lower levels of the beneficial bacteria *Akkermansia* and higher levels of pro-inflammatory *Proteobacteria* in the microbiome of young mice that received the fecal transfer (Fransen et al., 2017).

The microbiome also influences the response to respiratory pathogens via the gut-lung axis (Enaud et al., 2020). Gut microbes, in general, are protective against respiratory infection, as mice with a depletion of the microbiome have impaired immune responses and worse outcomes following bacterial or viral respiratory infection (Luján et al., 2010; Chen et al., 2011; Ichinohe et al., 2011; Fagundes et al., 2012; Schuijt et al., 2016). However, specific microbial populations in the gut can beneficially alter innate immune cell function in the lung. For example, colonization of the gut with segmented filamentous bacteria can improve the immune response to *S. pneumoniae* infection by promoting neutrophil clearance from the lung and resolution of inflammation (Felix et al., 2018).

As age is associated with changes in the microbiome and the gut-lung axis has been implicated in the response to respiratory pathogens, we designed this study to evaluate whether an impaired response to intranasal *S. pneumonia* infection parallels detrimental changes in the microbiome in a murine model of aging. We found that aged mice have increased morbidity and mortality after infection along with increased neutrophil infiltration into the lung. Furthermore, microbiome analysis revealed age specific expansion of the *Enterobacteriaceae* family of bacteria in the gut of aged *S. pneumoniae* infected mice which paralleled an increase of these gut-associated bacteria in the lung. These data provide important insight into a possible role of the gut-lung axis in age and infection.

## Methods

### Mice

Young and aged female BALB/cBy mice were obtained from the National Institute on Aging (NIA) colony (Charles River Laboratories, Wilmington, MA). Mice were housed at the University of Colorado Anschutz Medical Campus vivarium for a minimum of 2 weeks prior to any experimentation. Young mice were 4–5 months of age (similar to humans 25–30 years) and aged mice were 21–22 months of age (similar to humans >65 years) (Flurkey et al., 2007). All animal experiments were performed humanely under a protocol approved by the Animal Care and Use Committee of the University of Colorado Anschutz Medical Campus. Experiments were performed between the hours of 8–10 a.m. to avoid confounding factors related to circadian rhythms. Mice with tumors were removed from the study and mice losing more than 15% of their body weight were humanely euthanized.

### Infection

*S. pneumoniae* serotype 3 (ATCC 6303) infection was performed as previously described (Puchta et al., 2014). Briefly, glycerol stock was thawed in tryptic soy broth at 37°C, and incubated statically at 37°C/5% CO_2_ until the culture reached mid-log phase (OD 600 nm = 0.45–0.55). Bacteria were washed twice in sterile PBS and resuspended at an appropriate volume to yield approximately 10^5^ colony forming units (CFU) in 50 µl. Bacterial CFU were verified by culturing serial dilutions of the bacterial inoculate on Tryptone Soya Agar plates with 5% sheep blood (Fisher Scientific) at 37°C/5% CO_2_ for 18 h. Mice were anesthetized with an intraperitoneal injection of 12.5 mg/kg of ketamine and 1.25 mg/kg of xylazine (Webster Veterinary), and 50 ul of the prepared inoculum or sterile PBS was instilled intranasally. Mice were held vertically for 1 min to assist inoculum draining into the lungs. Blood CFU were determined by plating 10 ul of whole blood on Tryptone Soya Agar plates with 5% sheep blood (Fisher Scientific) at 37°C/5% CO_2_ for 18 h.

### Plethysmography

Respiratory function was measured using unrestrained whole-body barometric plethysmography (Buxco Research Systems) as described (Shults et al., 2015). Briefly, mice were allowed to acclimate in the sealed chamber for 5 min and the parameters of enhanced pause (penh), breathing frequency (breaths per minute) and tidal volume (ml) were recorded for 10 min by the manufacturer’s software (Buxco FinePointe).

### Lung Histology and Immunohistochemistry

Histological analysis and IHC staining of lungs was performed as previously described, with the following modifications (Patel et al., 1999). The left lung lobe was gravity inflated in 10% formalin and 5 µm paraffin-embedded sections were stained using hemoxylin and eosin (H&E) and scored in a blinded fashion by an experimental pathologist. Briefly, a semi-quantitative total injury score was determined for each tissue section by visual assessment of several pathological criteria including: the number and size of large inflammatory cell accumulations (0–6), the quantity of neutrophils in the airways (0–2), the presence of pigmented macrophages (0–1), the presence of peri-vascular inflammation (0–2) and edema (0–2), and the presence of alveolar debris (0–2). For immunohistochemistry, 8 µm sections were de-paraffinized and antigen retrieval was achieved by heating slides in a pressure cooker for 10 min in citrate buffer (Dako). Slides were placed in 3% H_2_O_2_ for 10 min, washed in PBS +0.1% Tween 20, and incubated for 20 min in 2.5% normal serum (Vector Laboratories). Sections were immersed in primary antibody for 1 h (Ly6G 1:250, BD Biosciences 551,459; *S. pneumoniae* 1:1,000; Novus Biologicals NB100-64502), washed, incubated with polymer reagent (Vector Laboratories) for 30 min and washed. Ly6G expression was assayed using 3,3′Diaminobenzidine and *S. pneumoniae* using alkaline phosphatase according to manufacturer’s protocol (Vector Laboratories). Slides were counterstained with Hematoxylin QS (Vector Laboratories), dehydrated in 99% isopropanol, and mounted with VectaMount medium (Vector Laboratories).

### Quantitative Real-Time PCR

RNA isolation and qRT-PCR was performed from snap frozen lung tissue as previously described (Curtis et al., 2019). Briefly, RNA was isolated using the RNeasy Mini kit (Qiagen) and converted to cDNA by reverse transcription using the iScript cDNA Synthesis kit (Bio-Rad). RT-PCR was performed on the QuantStudio 3 Real-Time PCR System (Thermo Fisher) using TaqMan qPCR Master Mix (Applied Biosystems) and TaqMan probes with the FAM reporter: *Ly6g* (Mm04934123_m1), *Cxcl1* (Mm04207460_m1), *Ccl2* (Mm00441242_m1), *Cxcl2* (Mm00436450_m), *Ccl5* (Mm01302427_m) and *Cxcr2* (Mm99999117_m) (ThermoFisher). Results were analyzed using the ΔΔCt algorithm (Livak and Schmittgen, 2001). *Gapdh* (Mm01143545_m1) with the VIC reporter (ThermoFisher) was used as and endogenous control.

### Measurement of Lung p38 Phosphorylation

Right lung lobes were collected in cold Hank’s buffered salt solution (HBSS; Gibco) containing 2 mg/ml Collagenase D and 0.1 mg/ml DNase I (Sigma-Aldrich) and kept on ice. Samples were dissociated using a GentleMACS machine (Miltenyi Biotec), incubated at 37°C/5% CO_2_ for 30 min, filtered through 70 µm nylon mesh filters, washed, and resuspended in Ammonium-Chloride-Potassium (ACK; Gibco) buffer to lyse red blood cells. After a PBS wash, cell pellets were lysed with Cell Lysis Mix and analyzed in duplicate wells for phosphorylated and total p38 MAPK according to manufacturer’s protocol (Invitrogen InstantOne ELISA kit).

### Lung Neutrophil Isolation

Bronchoalveolar lavage (BAL) was performed as previously described, with minor modifications (Shults et al., 2016). Briefly, lungs were aspirated multiple times with 1 ml of PBS using an 18-gauge catheter. Collected BAL fluid was pooled for each animal and red blood cells were lysed with ACK buffer. Viable cells were counted using Trypan blue exclusion. Gr-1^hi^Ly6G^+^ neutrophils were magnetically separated from total BAL cells using a Myeloid Derived Suppressor Cell Isolation kit (Miltenyi Biotec) according to manufacturer’s protocol.

### Bacterial PCR

Real time quantitative PCR (qPCR) was used to quantify bacterial DNA in the lungs of mice as described previously (Earley et al., 2015). Briefly, DNA was extracted from lung using a DNeasy Blood and Tissue Kit (Qiagen), following the manufacturer’s recommended protocol. qPCR for 18s and *S. pneumonaie* was performed using TaqMan Fast Advanced Mastermix (Applied Biosystems). The TaqMan 18s probe (4319413E, Applied Biosystems) and a custom CspA forward primer (cpsA-348F (5′-GCT​GTT​TTA​GCA​GAT​AGT​GAG​ATC​GA-3′), reverse primer (cpsA-415R (5′-TCC​CAG​TCG​GTG​CTG​TCA-3′) and probe (cpsA-TaqMan FAM 5′-FAM-AATGTTACGCAACTGACGAG-MGBNFQ1-3′) were used as previously described (Park et al., 2010). SYBR Green (Bio-Rad) in conjunction with forward and reverse primers synthesized by Integrated DNA Technologies were used to detect Pan bacteria (F: TCC​TAC​GGG​AGG​CAG​CAG​T, R: GGA​CTA​CCA​GGG​TAT​CTA​ATC​TT), *Escherichia coli* (F: CAT​GCC​GCG​TGT​ATG​AAG​AA, R: CGG​GTA​ACG​TCA​ATG​AGC​AAA), and *Enterobacteriaceae* (F: GTGCCAGCMGCCGCGGTAA, R: GCC​TCA​AGG​GCA​CAA​CCT​CCA​AG). Reactions were run on a QuantStudio 3 Real-Time PCR System (Applied Biosystems). Bacteria were quantified as a ratio of target gene/18s.

### Microbiome Analysis

Fecal bacterial profiles were determined by broad-range amplification and sequence analysis of 16S rRNA genes as previously described (Wheatley et al., 2020). Briefly, DNA was extracted using the QIAamp PowerFecal kit (Qiagen) and amplicons were generated using primers targeting the V3V4 variable region of the 16S rRNA gene. PCR products were normalized using a SequalPrep kit (Invitrogen), purified and concentrated using a DNAClean and Concentrator Kit (Zymo) and quantified using Qubit Fluorometer 2.0 (Invitrogen). Illumina paired-end sequencing was performed on the Miseq platform with versions v2.4 of the Miseq Control Software and of MiSeq Reporter, using a 600-cycle version 3 reagent kit. MicrobiomeAnalyst software (Dhariwal et al., 2017; Chong et al., 2020) was used for data display and analysis. Beta diversity was determined based on the Bray-Curtis Index and significant differences in beta diversity were assessed by PERMANOVA. Standard measures of alpha biodiversity, including richness (the number of OTUs per sample estimated by Chao1), community evenness (the uniformity of OTU distributions estimated by Shannon) and species diversity (Simpson) were estimated, and ANOVA was performed across the four groups for each diversity index. Differences in the relative abundances of individual OTUs between groups were assessed by nonparametric Mann-Whitney/Kruskal–Wallis tests. n = 8–10 mice per group from two identical repeat experiments.

### Other Statistical Analysis

For all non-microbiome related statistical analysis Prism 9 statistical analysis software (GraphPad) was used to perform the analysis. For comparison of two groups, an unpaired two-tailed Student’s *t* test was used. For comparison of more than two treatment groups a two-way ANOVA with Tukey’s multiple comparisons statistical test was used. A *p*-value of 0.05 or less was considered significant. Error bars indicate mean ± SEM.

## Results

### Infection With *S. pneumoniae* in Aged Mice Results in Increased Mortality, Impaired Lung Function and Heightened Bacterial Growth in the Lung

**FIGURE 1:**
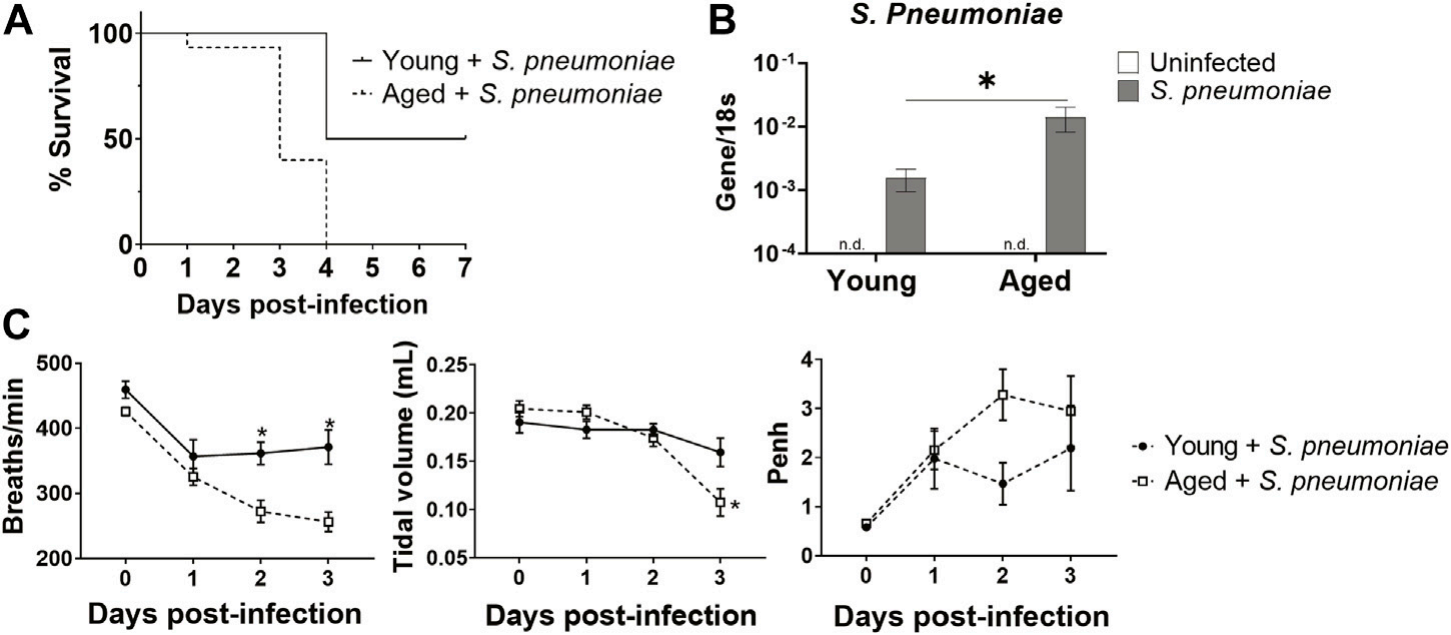
Aged mice have increased susceptibility to intranasal *S. pneumoniae* infection compared to young mice. **(A)** Survival curve for young and aged mice after intranasal infection with *S. pneumoniae.* *p < 0.05 by Log-rank test. n = 10–15 animals per group. **(B)** PCR for *S. pneumoniae* DNA in lungs isolated from uninfected or 24 h after *S. pneumoniae* infection in young and aged mice. Error bars indicate mean ± SEM. *p < 0.05 by *t* test. n = 3–15 mice per group. n. d. = not detected. **(C)** Respiratory functions of breath frequency (breaths/minute), tidal volume (ml) and enhanced pause (Penh) were measured in young and aged-infected animals by unrestrained whole-body plethysmography. *p < 0.05 by *t* test. n = 8–14 animals per group.

To begin to evaluate the effects of advanced age on *S. pneumoniae* infection, we infected young and aged mice with Serotype 3 *S. pneumoniae* intranasally and monitored survival for 7 days after infection (Figure 1A). Aged mice had 100% mortality at day 4 after infection, compared to 50% survival in the young group, mirroring the increased mortality rates observed in humans (Muder, 1998). Whole lung PCR for *S. pneumoniae* revealed a 10-fold increase (*p* < 0.05) in bacterial DNA in the lung of aged mice 24 h after infection, relative to the lung of young-infected animals, suggesting an impaired anti-bacterial response in the older group (Figure 1B). Next, we compared lung function in infected young and aged mice by performing whole body plethysmography daily for 3 days after infection. Aged mice had a 25% decrease (*p* < 0.01) in breath frequency compared to young mice by 48 h after infection (Figure 1C). In addition, there was an age-specific 52% reduction (*p* < 0.05) in tidal volume at 72 h after infection (Figure 1C). Airway responsiveness, as measured by an increase in Penh, was also elevated at 24 h in the aged mice, although this failed to reach significance. Taken together, these observations confirm that advanced age significantly alters the disease course of *S. pneumoniae* infection, with greater mortality and impaired lung function.

### Age-Related Exacerbation of *S. pneumoniae* Infection in Mice Is Associated With Increased Peri-vascular Lung Inflammation and Neutrophil Infiltration

**FIGURE 2:**
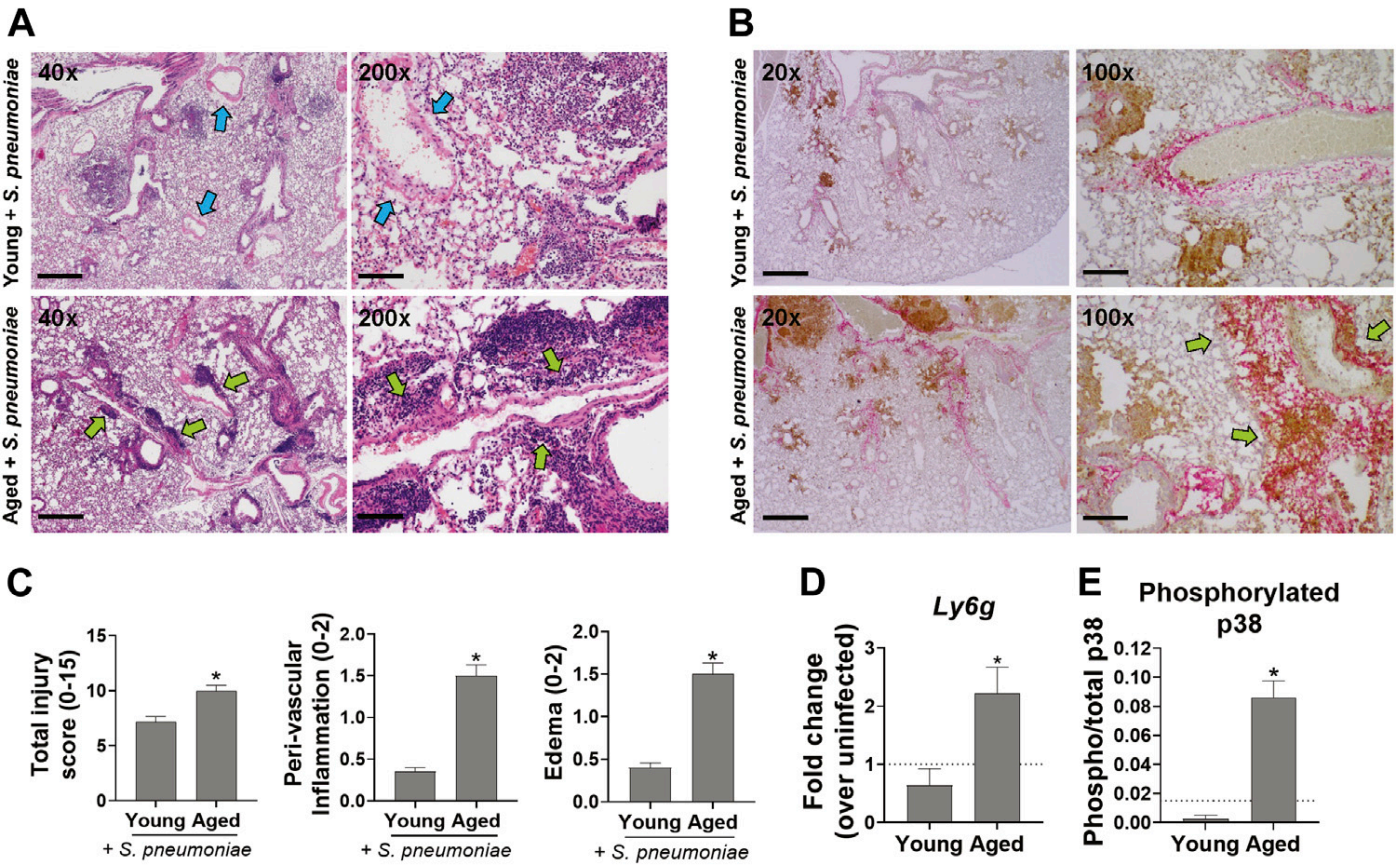
Aged mice have increased lung inflammation following infection with *S. pneumonia* compared to young mice. **(A)** Representative images of H&E stained lungs from 4-6 mice per group at 40x and 200x magnification are shown. Scale bars = 500 and 100 μM microns respectively. Blue arrows point to vessels with minimal peri-vascular inflammation. Green arrows point to regions with peri-vascular inflammation. **(B)** IHC staining of lungs for neutrophils (Ly6G, brown) and *S. pneumoniae* (pink). Representative images from 4–6 mice per group at 20x and 100x magnification are shown. Scale bars = 1000 and 200 μM respectively. Green arrows point to regions with extensive neutrophil infiltration and peri-vascular inflammation. **(C)** Bar graphs showing histologic scoring of the lungs. Data are presented as mean score for each group. *p < 0.05 compared to young-infected mice by *t* test. **(D)** qRT-PCR of *Ly6g* mRNA in whole lung tissue 24 h after infection with *S. pneumoniae*. *n* = 8–10 mice per group. Dashed line is mean level in young uninfected animals (n = 3). *p < 0.05 compared to young-infected mice by *t* test. **(E)** Whole lung lysates were analyzed for phosphorylated and total p38 MAPK by ELISA. Data are mean phosphorylated/total p38 in infected animals. n = 4-6 mice per group. Dashed line is mean ratio of young uninfected animals (n = 2). *p < 0.05 compared to young-infected mice by *t* test.

To determine if the increased mortality and impaired pulmonary function seen in infected aged mice was associated with excessive lung damage, when compared to young mice, lungs were harvested 24 h after infection, sectioned and stained with H&E for evaluation of pulmonary inflammation and edema. Leukocyte recruitment was apparent in and adjacent to the large airway mucosa in both young and aged mice at this timepoint (Figure 2A, blue arrows). However, leukocyte accumulation was more diffuse in the lungs of aged animals, including significantly greater peri-vascular inflammation (*p* < 0.0001) along with significantly more edema (*p* < 0.01), relative to younger mice (Figures 2A,C, green arrows). Immunohistochemical staining for the neutrophil marker Ly6G showed an overall increase in neutrophil accumulation in lungs from infected aged mice that was not seen in the lungs of young-infected animals. In young mice, the neutrophils were predominantly adjacent to the airways, while in aged mice these cells accumulated around both the alveoli and near the vasculature (Figure 2B, brown stain, green arrows). In addition, mRNA levels of *Ly6g* were 4.5-fold higher (*p* < 0.05) in the lungs of aged-infected mice compared to those of young-infected mice (Figure 2D). The increase in neutrophils paralleled increased activation of p38 MAPK (mitogen-activated protein kinase) in the lung of aged mice indicating increased activation of pro-inflammatory signaling in the lungs (Figure 2E) (Qian et al., 2016). We saw no obvious changes in macrophage numbers in the lungs of infected mice at this timepoint (data not shown). These data reveal an exacerbation of neutrophil-associated inflammation in the lungs of aged-infected mice that is not observed in young mice, which could explain the impaired lung function and increased mortality with age. Relative to younger infected mice, aged mice also had more staining for *S. pneumoniae* in their lungs (Figure 2B, pink stain), which is in agreement with the data from Figure 1B showing higher levels of *S. pneumoniae* DNA in the lungs of aged versus young mice. Thus, despite the increased neutrophil infiltration, aged mice are unable to sufficiently control *S. pneumoniae* within the lung.

### Age-specific Increased Chemokine Gene Expression in Lung Neutrophils After *S. pneumoniae* Infection

**FIGURE 3:**
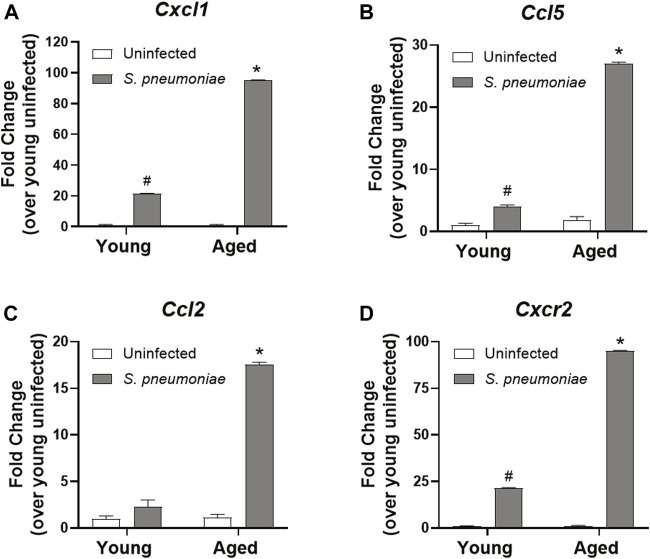
Lung neutrophils from aged mice have increased chemokine expression following *S. pneumoniae* infection compared to young mice. qRT-PCR for *Cxcl1* **(A)**, *Ccl5* **(B)**, *Ccl2* **(C)** and *Cxcr2* **(D)** in neutrophils isolated from the lungs of young and aged uninfected or *S. pneumoniae*-infected mice. Data are presented as mean fold change over young uninfected lung neutrophils and significant changes were determined by ANOVA. *p < 0.05 compared to all other groups. ^#^ *p* < 0.05 compared to uninfected groups only. n = 3-6 mice per group.

Despite the increased number of neutrophils in the lung, we failed to observe a rise in the expression of the pro-inflammatory cytokines, TNFα, IL1β or IL-6, in the lung of aged mice after infection, when compared to the lungs of young-infected mice (data not shown). Therefore, we next evaluated the expression of chemokines in our model to determine if age-related changes in immune cell recruitment could help explain the increased neutrophil infiltrate. To accomplish this, we isolated Ly6G^+^ neutrophils from the bronchoalveolar fluid of *S. pneumoniae* infected young and aged mice 24 h after infection and examined chemokine expression. Neutrophils from aged-infected mice expressed 4.5-fold higher levels of mRNA for the neutrophil chemokine (C-X-C motif) ligand 1, CXCL1 (KC) compared to those from young-infected mice (*p* < 0.001) (Figure 3A). CXCL1 has been shown to be critical for the homing of neutrophils to the lung in pneumonia (Paudel et al., 2019), suggesting that chemokine production by neutrophils themselves is likely contributing to additional cellular recruitment to the lung in age. We also observed age-dependent increases in expression of chemokines involved in recruiting other immune cells. (C-C motif) ligand 5 (CCL5; also known as RANTES), which is important for the direct recruitment of multiple cell types, including T cells, eosinophils and basophils, but can also induce indirect recruitment of neutrophils via activation of macrophages (Hwaiz et al., 2015), was increased ∼6 fold from young mice after infection (*p* < 0.001) (Figure 3B). mRNA encoding (C-C motif) ligand 2 (CCL2 or monocyte chemoattractant protein-1 (MCP-1)), a chemokine responsible for recruiting monocytes to peripheral tissues (Deshmane et al., 2009) was transcriptionally upregulated 7.5-fold in aged mice, compared to young mice (*p* < 0.001) (Figure 3C). Lung neutrophils from aged-infected mice had 4.5-fold higher expression of CXCR2, the receptor for CXCL1, relative to lung neutrophils from young mice (*p* < 0.001) (Figure 3D) and may, therefore, be primed for increased recruitment into tissues in the aged animals by CXCL1 or other chemokines.

### Intranasal Infection With *S. pneumoniae* Infection Leads to Alterations in the Fecal Microbiome in Both Young and Aged Mice

**FIGURE 4:**
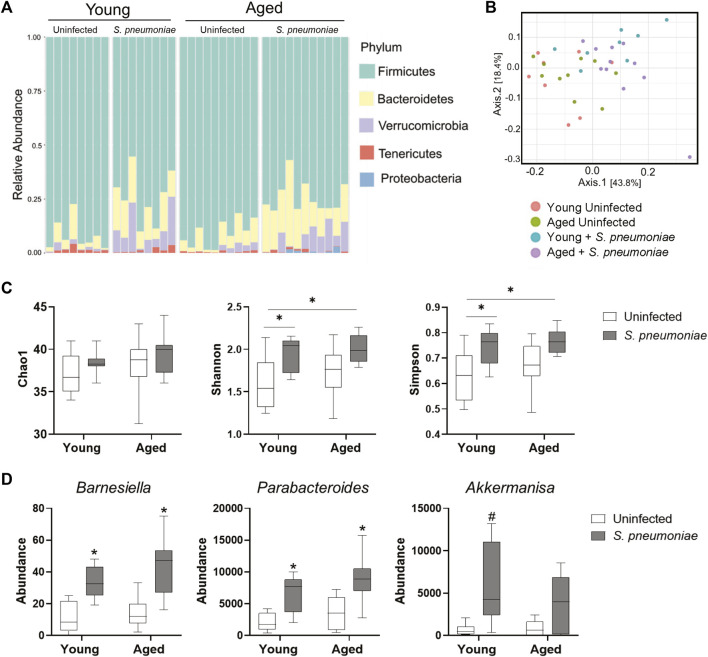
*S. pneumoniae* infection is associated with alterations in the fecal microbiome in both young and aged mice. **(A)** Schematic representation of bacterial phyla in the fecal microbiota composition across young and aged mice pre- and post-infection with *S. pneumoniae*. Bars represent relative abundances of the 5 most abundant phyla for each individual mouse. **(B)** PCoA plot showing clustering of beta diversity of the fecal bacterial populations between the different treatment groups. **(C)** Measures of alpha-diversity within each fecal microbiome: richness (Chao1), evenness (Shannon) and diversity (Simpson). *p < 0.05 by ANOVA. **(D)** Results of Kruskal-Wallis tests of relative abundance of specific taxa across all four treatment groups. *p < 0.05 compared to both young and aged uninfected. #*p* < 0.05 compared to young uninfected. n = 8–10 mice per groups.

It has been demonstrated that the gut microbiome can alter the neutrophilic response to *S. pneumoniae* intranasal infection by promoting neutrophil clearance from the lung (Felix et al., 2018). Therefore, to evaluate the effects of age and *S. pneumoniae* infection on the gut microbiome in our model, we performed 16s rRNA sequencing of fecal pellets from young and aged mice at 24 h prior to and at 24 h after infection. Fecal bacterial profiling revealed broad alterations of bacterial phyla in both young and aged mice after infection with *S. pneumoniae* (Figure 4A). Comparison of beta diversity between treatment groups indicated a small but significant shift in the species diversity in the infection groups compared to the uninfected groups (*p* < 0.05) (Figure 4B). Analysis of three common measures of alpha biodiversity, Chao, Shannon and Simpson, showed no effect of either age or infection on bacterial richness (Chao), but *S. pneumoniae* induced a significant change in bacterial community evenness (Shannon, *p* < 0.01) and diversity (*p* < 0.01) compared to the age-matched uninfected group, indicating an infection-induced expansion of bacterial species within the microbiome of young and aged mice (Figure 4C). Comparison of individual taxa pre- and post-infection showed a significant infection-dependent increase in two distinct genera belonging to the phylum *Bacteroidetes*, *Barniesiella* (*p* < 0.05) and *Parabacteroides* (*p* < 0.01) in both the young and the aged mice compared to uninfected microbiome (Figure 4D). Infection in young, but not aged, mice also led to increased expansion of the beneficial bacteria *Akkermansia* compared to the young uninfected group (*p* < 0.05). Taken together, these data demonstrate that intranasal infection with *S. pneumoniae* can markedly alter the gut microbiome in both young and aged mice.

### *S. pneumoniae* Infection Induces Age-specific Increases in *Enterobacteriaceae* in the Gut and the Lung

**FIGURE 5:**
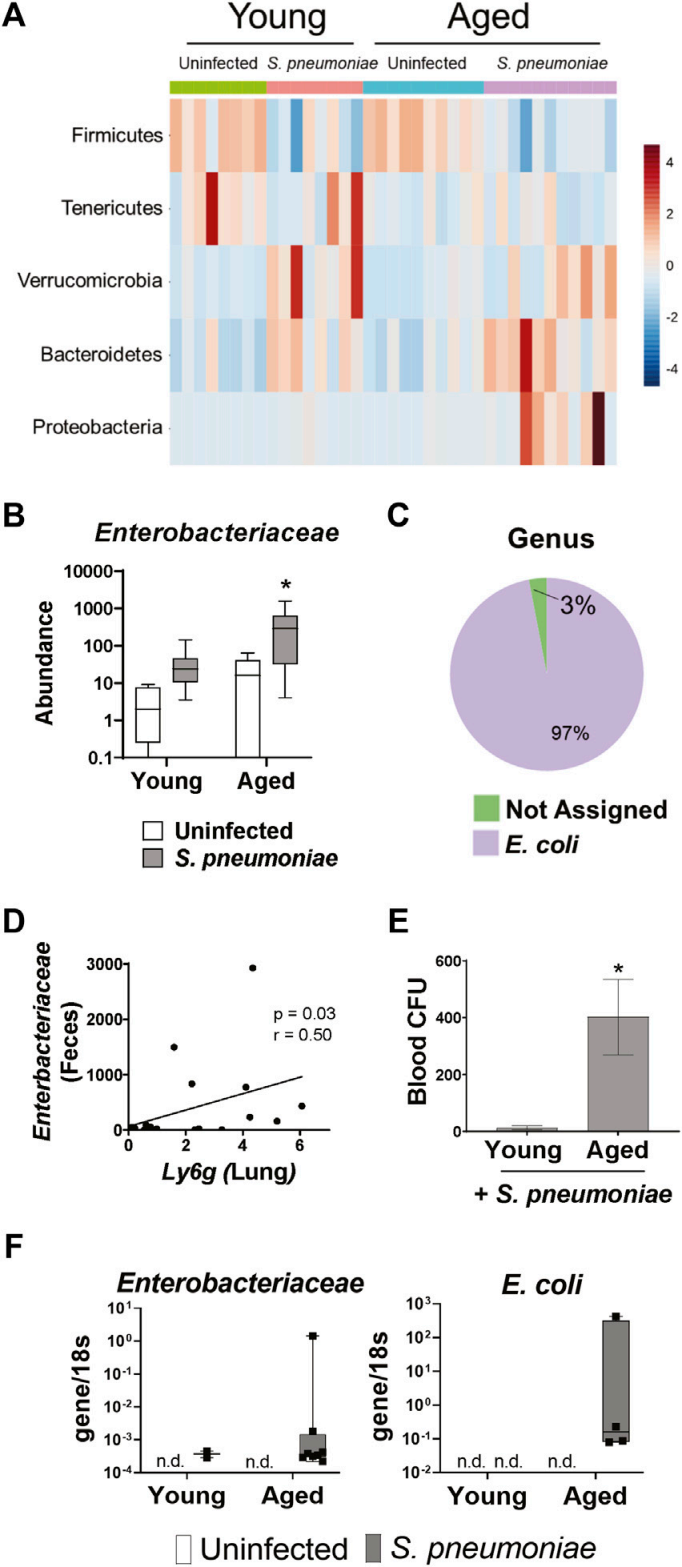
*S. pneumoniae* infection induces an age-specific rise in *Enterobacteriaceae.* **(A)** Heat map illustrating the relative abundance of the top five bacterial phyla in the feces of young and aged mice. **(B)** Bar graph showing mean abundance of *Enterobacteriaceae* in the feces from the indicated treatment group. *p < 0.05 from all other groups by ANOVA with post-hoc Tukey’s test. **(C)** Pie chart showing the mean abundance of different bacterial genre within the *Enterobacteriaceae* population in aged mice following *S. pneumoniae* infection. n = 8–10 mice per group. **(D)** Correlation analysis between fecal *Enterobacteriaceae* and expression of *Ly6g* mRNA in whole lung tissue 24 h after infection with *S. pneumoniae*. Spearman’s rank correlation test was used. **(E)** Quantification of blood CFU in young and aged mice 48 h after infection. *n* = 10–14 mice per group. *p<0.05 from all other groups by t test. **(F)** PCR for *Enterobacteriaceae* and *E. Coli* DNA in lungs isolated from vehicle or infected young and aged mice 24 h after infection. Graphs represent transcript over *18s*. n = 3–10 mice per group. n. d. = not detected.

We next wanted to determine if the *S. pneumoniae-*induced gut microbiome changes differed between young and aged mice. Analysis of individual bacterial phyla showed an increase in *Proteobacteria* in the feces following infection in the aged, but not young mice (*p* < 0.05) (Figure 5A). *Proteobacteria* is a major phylum of Gram-negative bacteria in the gut microbiome that has been associated with dysbiosis in a number of disease models (Zeng et al., 2017). Further analysis of the *Proteobacteria* population revealed an 11-fold increase (*p* < 0.05) in the relative abundance of the *Enterobacteriaceae* family in the aged-infected feces, compared to young-infected mice (Figure 5B). The *E. coli* genus accounted for >95% of the *Enterobacteriaceae* in the gut of these mice (Figure 5C).

To determine if expansion of these potentially pathogenic symbiotes in the gut microbiome parallels changes in the lung we performed a correlation analysis of fecal *Enterobacteriaceae* and lung expression of *Ly6g* mRNA and found a significant positive correlation between the two variables (Figure 5D). Furthermore, aged mice had significantly higher levels of bacteria in the blood, compared to young mice, following infection (Figure 5E). Therefore, we performed PCR for *Enterobacteriaceae* and *E. coli* DNA on the lungs of young and aged mice with and without *S. pneumoniae* infection. We were unable to detect any DNA for either *Enterobacteriaceae* or *E. coli* in the lungs of uninfected young or aged mice, confirming that *Enterobacteriaceae* is not a prominent bacterial family in the healthy murine lung, regardless of age (Figure 5F). *Enterobacteriaceae* was, however, detected in the lungs of both aged and young mice 24 h after *S. pneumoniae* infection. Interestingly, we found *Enterobacteriaceae* in significantly more of the aged-infected than young-infected animals (25% v. 80%, *p* < 0.05) (Table 1). Furthermore, *E. coli* DNA was only found in the lungs of aged-infected mice, with 40% of the mice having detectable amounts in the lung at 24 h after infection (Figure 5F; Table 1). These data confirm the occurrence of age-specific bacterial dysbiosis in both the gut and the lung following infection with *S. pneumoniae*, suggesting changes in the gut-lung axis could be contributing to the altered response to this pathogen in aged mice.

**TABLE 1:** Number of mice positive for *Enterbacteriaceae* and *E. coli* DNA in the lungs 24 h after infection. (%) *p<0.05 compared to young *S. pneumoniae* infected mice by Pearson’s chi-squared test.

Bacteria	Young		Aged	
	Uninfected N (%)	*S. pneumoniae* N (%)	Uninfected N (%)	*S. pneumoniae* N (%)
*Enterobacteriaceae*	0/3 (0)	2/8 (25)	0/6 (0)	8/10 (80)*
*E. coli*	0/3 (0)	0/8 (0)	0/6 (0)	4/10 (40)*

## Discussion

Infection with *S. pneumoniae* is one of the most common causes of pneumonia in people over the age of 65 (Ewig et al., 2012) and advanced age is an independent risk factor for poor outcomes following infection (Loeb et al., 1999). In this study, we found that aged BALB/c mice exhibit similar heightened susceptibility to pneumonia and increased mortality compared to their younger counterparts by 72 h after infection with serotype 3 *S. pneumoniae*. In addition, analysis of whole-body plethysmography revealed a clear delineation of lung function between aged and young groups at the 48 h timepoint. Analysis of lung histology 24 h after infection, which has been shown to correlate with early neutrophil infiltration into the lungs of young mice (Bergeron et al., 1998), showed that aged mice had increased accumulation of neutrophils in the lung, especially surrounding the vasculature, compared to their younger counterparts. This age-related increased neutrophilic response in the lung is in agreement with clinical studies which found higher numbers of lung neutrophils in *S. pneumonia* infected elderly patients, compared to infected young patients (Menter et al., 2014). In murine studies, pulmonary levels of pro-inflammatory cytokines, including IL-6, IL1-β and TNFα, increase after *S. pneumoniae* infection (Bergeron et al., 1998), and have been shown to correlate with the severity of disease (Takashima et al., 1997; Dallaire et al., 2001). Baseline levels of these cytokines are also increased in age and are thought to contribute to a number of age-related diseases (Michaud et al., 2013). Interestingly, in our study aged and young mice had a similar increase in mRNA encoding IL-6, IL1-β and TNFα in the lung after infection indicating that age-related alterations in pro-inflammatory cytokines, at least at the transcriptional level, are not driving the heightened pulmonary response in our model. However, increased expression of genes encoding chemokines, including the neutrophil recruiting chemokine CXCL1, was observed in the aged mice. It is of interest that p38 MAPK activity is important in CXC chemokine production in the lung and recruitment of neutrophils to the pulmonary microvasculature (Zhang et al., 2012) since we found increased activation of this pathway in the lungs of aged mice. Further exploration of this pathway in future studies is warranted.

Neutrophils function as efficient phagocytes and display potent antimicrobial activity via production of reactive oxygen species, antimicrobial peptides and serine proteases such as neutrophil elastase (NE) and are therefore critical in initial immune response to pneumonia. However, these effector functions are non-specific and, especially in the case of NE, can also cause damage to the surrounding tissue if not properly regulated (Weiss, 1989; Fox et al., 2013). It is plausible that, in our aged-infected mice, excessive neutrophil accumulation within the lung is exacerbating tissue damage and future studies utilizing neutrophil depletion or CXCL1 neutralization in our model would confirm this hypothesis. Alternatively, other studies show that advanced age results in reduced ability of neutrophils to phagocytose bacteria and produce reactive oxygen species (Wenisch et al., 2000; Bhalla et al., 2020) ***.*** The fact that we saw higher levels of *S. pneumoniae* in aged-infected lungs compared to young-infected lungs, despite the increased number of neutrophils, would support the premise that the neutrophils occupying the lung in age are not as efficient at killing the bacteria.

The mechanisms behind what causes age-related changes in innate immunity is an area of great interest and a growing number of studies point towards a role of the gut microbiome. For example, as noted above, transfer of the fecal microbiome of aged mice into young mice induced systemic inflammation and was associated with lower levels of *Akkermansia* and higher levels of *Proteobacteria* in young mice after transfer (Fransen et al., 2017). Interestingly, we found that intranasal infection with *S. pneumoniae* differentially alters the gut microbiome in young and aged mice with an age-specific expansion of *Proteobacteria*, notably the *Enterobacteriaceae* family. *Enterobacteriaceae*, which includes symbionts *Escherichia coli* and *Shigella*, are a large family of Gram-negative facultative bacteria. In the healthy gut, *Enterobacteriaceae* exists at low levels close to the mucosal epithelium (Zeng et al., 2017). However, the *Enterobacteriaceae* family members contain potent pro-inflammatory microbe-associated molecular patterns (MAMPs), such as lipopolysaccharide (LPS), and overgrowth of this family of bacteria in the gut has been linked to a number of diseases, including obesity and inflammatory bowel disease (Zeng et al., 2017). It remains to be established how intranasal infection with *S. pneumoniae* induces changes in the gut microbiome. It is feasible that altered food consumption or other metabolic changes in aged mice following infection could be influencing the diversity of the microbiome (Sheflin et al., 2017). However, inflammation in the gut seems to be particularly conducive to expansion of aerobic bacteria, especially the *Enterobacteriaceae* family (Lupp et al., 2007). It is unlikely that, in our model, the changes in the microbiome result from direct inflammatory effects of *S. pneumonia* in the gut as we were unable to detect any *S. pneumonia* sequences in the feces of any of the treatment groups. Instead, it is likely that increased systemic inflammation in aged-infected mice is contributing to the observed infection-induced dysbiosis.

Although not directly addressed in this study, it is possible that the observed expansion of *Enterobacteriaceae* in aged mice leads to a breakdown of the intestinal barrier as *E. coli* strains have been shown to directly disrupt the epithelial layer (Wine et al., 2010). This would allow for passage of gut MAMPs and bacteria into the lung, resulting in an exacerbation of pulmonary inflammation. Indeed, expansion of *Enterobacteriaceae* in the lung has also been observed in patients with Acute Respiratory Distress Syndrome (ARDS) (Dickson et al., 2016). In our study elevated fecal levels of *Enterobacteriaceae* correlated with increased neutrophil gene expression in the lung. Furthermore, aged mice had elevated levels of bacteria in the blood and *E. coli* was detected in the in the lungs of our aged, but not young-infected mice, suggesting an age-specific alteration in the gut–lung axis following *S. pneumoniae* infection. However, the possibility that infection is directly altering the lung microbiome remains (Thevaranjan et al., 2016). It is of note that age alone is associated with increased dysbiosis and impaired gut barrier function (Thevaranjan et al., 2017) and our lab has shown that aged mice are more susceptible to a breakdown in the intestinal barrier after other systemic insults (McMahan et al., 2021; Najarro et al., 2022). Alternatively, differential production of metabolites by the gut microbiome in aged-infected mice could also be influencing the lung. Bacterial metabolites such as short-chain fatty acids and bile acids have been shown to participate in the gut-lung axis and influence lung immunity (Budden et al., 2017) and future studies exploring these pathways are warranted.

In conclusion, we show here that intranasal infection with *S. pneumoniae* results in an age-specific exacerbation of neutrophilic infiltration into the lung, dysbiosis of the gut microbiome and accumulation of gut-specific bacteria in the lung. These microbial changes could contribute to an altered gut-lung axis in response to infection in age. The strain of *S. pneumoniae* used for infection in these studies, ATCC 6303, is highly virulent in mice and it will be of interest to determine if other virulent serotypes with decreased lethality in mice, including serotypes 2 and 4 (Lim et al., 2007), would also elicit similar age-related changes in the microbiome. In addition, future studies aimed at altering the microbiome in aged mice will provide critical data for addressing the age-related increased susceptibility to pneumonia.

## Data Availability

The datasets presented in this study can be found in online repositories. The names of the repository/repositories and accession number(s) can be found below: NCBI—PRJNA799655.
